# The role of a novel magnetic resonance imaging sequence in the evaluation of the fetal skeleton: a pilot study

**DOI:** 10.1590/0100-3984.2017.0100

**Published:** 2018

**Authors:** Renata do Amaral Nogueira, Heron Werner Júnior, Pedro Daltro, Glaucia Macedo Lima, Adauto Dutra Barbosa, Edward Araujo Júnior

**Affiliations:** 1 Clínica de Diagnóstico Por Imagem (CDPI), Rio de Janeiro, RJ, Brazil.; 2 Department of Maternal-Infant Care, Universidade Federal Fluminense (UFF), Niterói, RJ, Brazil.; 3 Department of Obstetrics, Escola Paulista de Medicina da Universidade Federal de São Paulo (EPM-Unifesp), São Paulo, SP, Brazil.

**Keywords:** Fetus, Bone diseases, developmental, Magnetic resonance imaging, Feto, Doenças do desenvolvimento ósseo, Ressonância magnética

## Abstract

**Objective:**

We aimed to study the role of magnetic resonance imaging (MRI), including a novel MRI sequence-the modified volumetric interpolated breath-hold examination (VIBE)-in the characterization of the fetal skeleton. This novel sequence was useful for reconstructing three-dimensional images of the skeleton.

**Materials and Methods:**

We enrolled 22 pregnant women whose fetuses had shown congenital abnormalities on ultrasound examinations. The women underwent prenatal fetal MRI in a 1.5-T scanner with a T2-weighted modified VIBE sequence. Three-dimensional reconstructions of the fetal skeleton were performed manually on the instrument itself or via an interactive pen-tablet workstation.

**Results:**

Three-dimensional reconstructions of the fetal skeleton were performed after the acquisition of modified VIBE MRI sequences, and it was possible to characterize the fetal skeleton in all MRI examinations.

**Conclusion:**

A detailed evaluation of the three-dimensional reconstructions of fetal skeleton performed after acquisition of a modified VIBE MRI sequence allowed a full characterization of the skeleton. However, improvements to the proposed sequence should be addressed in future studies.

## Abstract (pt)

**Objetivo:**

O objetivo deste estudo foi demonstrar o papel da ressonância magnética (RM) incluindo uma nova sequência chamada de *volumetric interpolated breath-hold examination* (VIBE)) modificada que permita a reconstrução tridimensional para avaliação do esqueleto fetal.

**Materiais e Métodos:**

Foram analisadas 22 mulheres grávidas portando fetos com anomalias congênitas, não exclusivamente esqueléticas, as quais foram observadas em exame de ultrassonografia prévia. Para a aquisição das imagens de RM utilizou-se aparelho 1,5 T com a sequência VIBE modificada, ponderada em T2. Reconstruções tridimensionais foram feitas, manualmente, no próprio aparelho ou em uma estação de trabalho *pen tablet* interativo.

**Resultados:**

Foi possível demonstrar a reconstrução tridimensional do esqueleto fetal por RM, com base em uma aquisição volumétrica da sequência VIBE modificada em todos os exames de RM do esqueleto fetal.

**Conclusão:**

Uma avaliação detalhada do esqueleto fetal pelas reconstruções tridimensionais realizadas após a aquisição da imagem usando sequência de RM VIBE modificada permitiu uma avaliação completa do esqueleto fetal, entretanto, as melhorias nessas sequências devem ser o foco de estudos futuros.

## INTRODUCTION

Magnetic resonance imaging (MRI) is typically used as a complementary imaging tool when congenital abnormalities are detected during prenatal ultrasound. Fetal MRI facilitates accurate diagnoses because it provides important anatomical information that can be helpful in planning prenatal and postnatal early care, thereby reducing perinatal morbidity and mortality^(1)^.

Developments and modifications in the hardware and software employed in MRI have improved its diagnostic accuracy in fetal studies. Several studies have emphasized the benefit of MRI in evaluating congenital anomalies of the brain and lungs, as well as complex syndromes, in the fetus^(1-3)^. However, few studies have investigated the contribution of MRI to the diagnosis of skeletal abnormalities of the fetus^(4-8)^.

Skeletal dysplasia has an incidence of approximately 2 cases per 10,000 live births; it is fatal in approximately 50% of the affected infants^(9)^. The condition can occur in isolation or in combination with genetic syndromes. Prenatal diagnosis (of the fetus) is essential for proper genetic counseling, prognosis, and postnatal management.

The aim of this study was to evaluate the feasibility of using a modified MRI technique that employs a T2-weighted three-dimensional (3D) gradient-echo volumetric sequence for the evaluation of the fetal skeleton.

## MATERIALS AND METHODS

This was a retrospective, cross-sectional study based on data related to pregnant patients who were referred for fetal MRI as part of their clinical care after abnormalities were identified during routine ultrasound examinations. The period of the study was from March 2012 to March 2013. The local institutional review board approved the study, and the need for written, informed consent was waived because of the retrospective nature of the study.

The inclusion criteria were as follows: singleton pregnancy; suspicion of fetal congenital anomaly for which the diagnosis was not conclusive on ultrasound examination; and gestational age > 22 weeks, as determined from the date of onset of the last menstrual period and confirmed by an ultrasound examination performed within the first 16 weeks of pregnancy. Women who were claustrophobic were excluded, as were those in whom oligohydramnios or obesity (body mass index > 30 kg/m^2^) made it difficult to interpret the ultrasound images.

The MRI examinations were performed in a 1.5-T scanner (Magnetom Aera; Siemens Healthineers, Erlangen, Germany) and were guided by a multidisciplinary team. A surface coil was placed on the abdomen of the patients, who were positioned in the supine or left lateral position, whichever was more comfortable. The following protocol was used: T2-weighted half-Fourier single-shot turbo spin-echo sequences (repetition time/echo time [TR/TE] = 1000/140 ms, field of view = 300-200 mm, and acquisition matrix = 256 × 256) with a slice thickness of 4 mm, an acquisition time of 18 s, and the acquisition of 40 slices in the axial, coronal, and sagittal planes of the fetus; 3D true fast imaging with steady-state precession (TR/TE = 3.16/1.4 ms and isotropic voxel size = 1.5 × 1.5 × 1.5 mm), with an acquisition time of 23 s and the acquisition of 96 slices; and modified volumetric interpolated breath-hold examination (VIBE) sequences (TR/TE = 11.0/9.53 ms, flip angle = 4°, and field of view = 380 mm), with a slice thickness of 2 mm, the acquisition of 60 slices, and an interslice gap of 0 mm. Neither intravenous contrast nor sedation was used in any of the cases.

**Figure 1:**
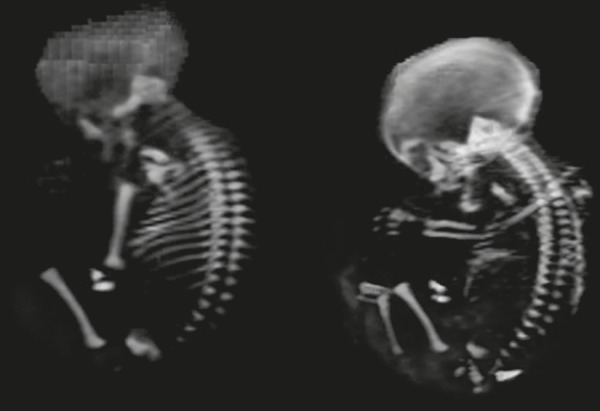
Fetus at 27 weeks of gestation. Evaluation of a skeleton after VRT 3D reconstruction.

**Figure 2:**
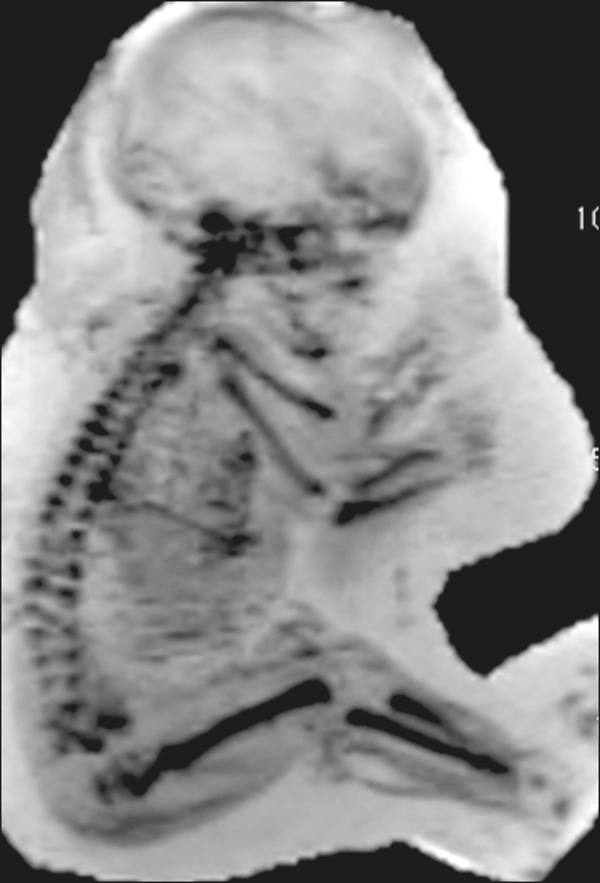
Fetus at 34 weeks of gestation. Evaluation of a section of the skeleton without abnormalities after image reformatting with MinIP.

**Figure 3:**
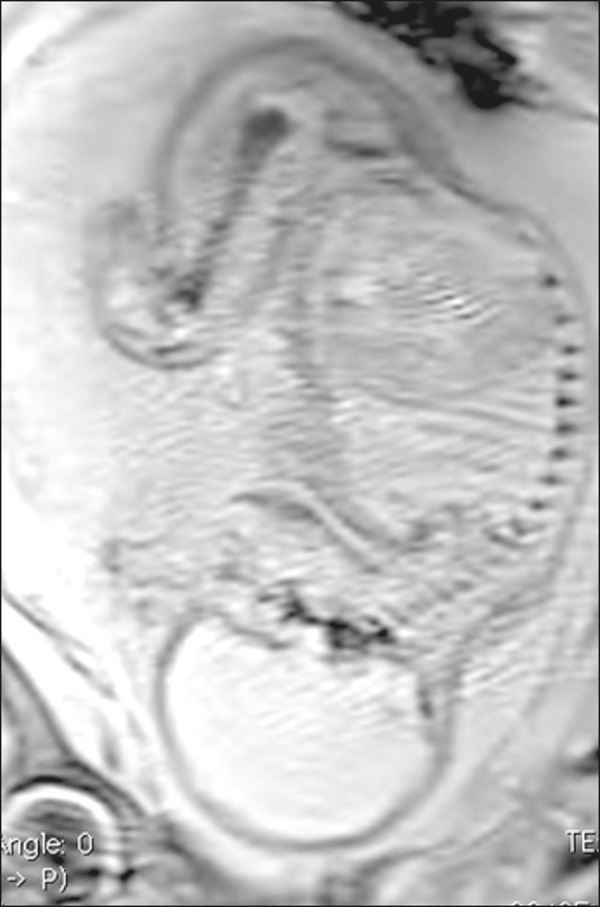
Fetus at 28 weeks of gestation. Sagittal modified VIBE sequence.

**Figure 4:**
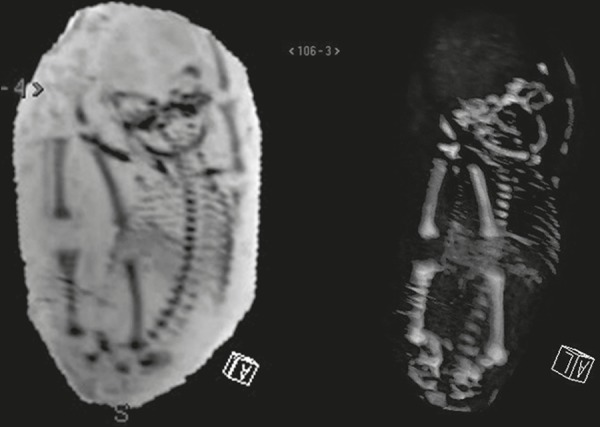
Fetus at 28 weeks of gestation. Image reformatting with MinIP and 3D VRT.

**Figure 5:**
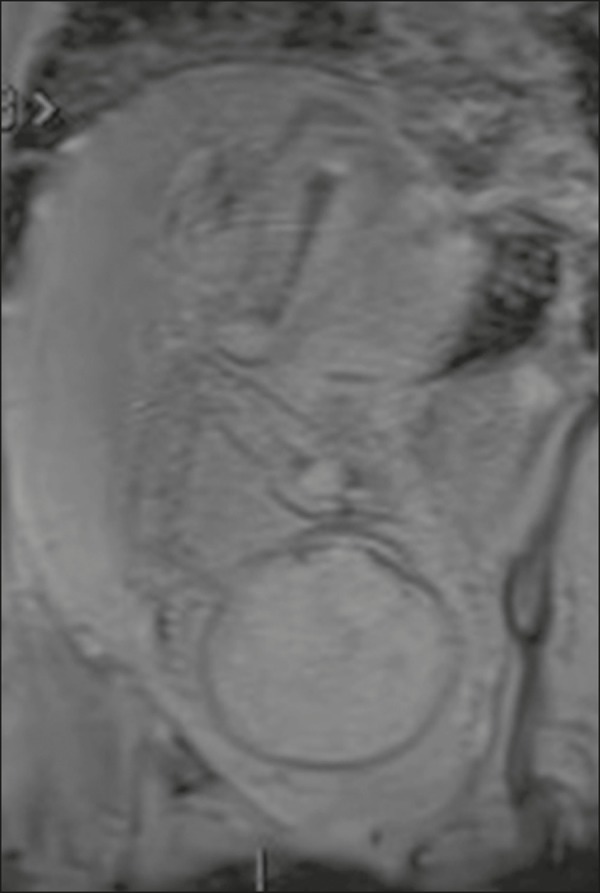
Fetus at 33 weeks of gestation. Sagittal modified VIBE sequence.

The 3D images of the fetal skeletons were analyzed after data had been acquired and subsequently reconstructed by an MRI technician on a workstation and the Aera platform (Siemens Healthineers) using the minimum intensity projection (MinIP), 3D maximum intensity projection (MIP), and 3D volume rendering technique (VRT) editor imaging tools (Figures 1-5). After the images had been acquired, the fetal skeleton was evaluated by three readers (two pediatric radiologists and one obstetrician, all of whom were experienced in fetal imaging).

## RESULTS

The study group included 22 female patients with a mean age of 31.3 ± 7.29 years (range, 15-43 years), and the mean gestational age of the fetuses was 29.3 ± 3.39 weeks (range, 22-35 weeks). We were able to evaluate the fetal skeleton via 3D reconstructions in all of the MRI examinations. Table 1 shows the characteristics of the sample, including fetal diagnosis, maternal age, and gestational age.

**Table 1:** Maternal age, gestational age, and fetal abnormalities among pregnant women undergoing MRI with modified VIBE sequences.

Case	Maternal age (years)	Gestational age (weeks)	Skeletal dysplasia of the fetus
1	15	34	Absent
2	37	33	Absent
3	34	31	Clubfeet
4	34	30	Absent
5	36	28	Absent
6	29	27	Absent
7	37	29	Absent
8	24	30	Absent
9	36	33	Absent
10	28	22	Absent
11	30	25	Fusion defect of the posterior arch of 6th lumbar vertebra
12	34	35	Absent right fibula, hypoplastic left fibula, and clubfeet
13	39	28	Absent
14	39	34	Absent
15	43	28	Absent
16	31	30	Absent
17	26	27	Congenital clubfoot
18	24	25	Long bone length at the 5th percen- tile for gestational age
19	17	26	Absent
20	34	33	Absent
21	38	28	Absent
22	24	29	Absent

In our sample, there were no cases of maternal obesity (body mass index > 30 kg/m^2^) or oligohydramnios (amniotic fluid index < 5 cm). Each MRI examination was completed within 30 min, and the acquisition of the modified VIBE sequence did not significantly lengthen the examination, the mean duration of the modified VIBE sequences being 18 s.

## DISCUSSION

A review of the literature showed that substantial advances have been made in the use of MRI to evaluate the fetal skeleton. Although not a substitute, MRI complements ultrasound because it is not limited by the fetal position, maternal obesity, or oligohydramnios. However, the accuracy of both methods can be slightly reduced if there is pronounced fetal movement or severe oligohydramnios.

The multiplanar reconstruction of MRI images enabled the complete evaluation of the fetus and contributed to the characterization of the fetal skeleton. Despite the small size of our sample, image reconstruction of the skeleton allowed the identification of defects in the long bones of the fetus, and this technique provided good resolution, thus allowing the definitive diagnoses to be made. Nemec et al.^(4)^ characterized the fetal skeleton using other MRI techniques, such as the acquisition of echo-planar imaging, thick-slab T2-weighted, and dynamic steady-state free precession sequences, and the characterization of all the parts of the fetal skeleton achieved with those sequences was comparable to that obtained with the modified VIBE sequences. We found that MRI with a modified VIBE sequence was more useful than was 3D ultrasound because the latter depends on the position of the fetus. The main goal of the present study was to propose a new MRI sequence for the diagnosis of skeletal dysplasia in fetuses.

We believe that our study has shown that the use of the modified VIBE sequence in MRI examinations creates the possibility of reconstructing the 3D images for the complete characterization of the fetal skeleton. However, this was a pilot study, and the evaluation of the hands and feet of the fetus continues to be a challenge. Unlike previously examined sequences^(10-12)^, which displayed only certain parts of the fetal skeleton, the modified VIBE sequences allowed the depiction of the entire fetal skeleton.

Previous studies have described a sequence that allows complete 3D evaluation-the thick-slab T2-weighted sequence^(11)^. The thick-slab T2-weighted sequence differs from the modified VIBE sequence in that a single acquisition is performed in 1 s and the image is reproduced by simulating a 3D evaluation. However, the modified VIBE sequence performs a true 3D analysis and has some advantages over 3D computed tomography and 3D ultrasound in the characterization of the entire skeleton. In comparison with 3D ultrasound, MRI is not limited by excess adipose tissue, oligohydramnios, or fetal movements. In comparison with 3D computed tomography, MRI with the modified VIBE sequence does not involve exposure to ionizing radiation^(12,13)^.

As previously mentioned, including the modified VIBE sequence for the characterization of the fetal skeleton did not significantly increase the duration of the MRI examination, because the estimated mean duration of the proposed sequence was 18 s, the total time of the examination being 30 min. It has been established that skeletal anomalies of the fetus can occur in isolation or in combination with chromosomal defects^(14)^, such anomalies being diagnosed in the prenatal period through the use of imaging methods and genetic testing^(15)^. Making the diagnosis in the prenatal period allows appropriate counseling of the parents regarding the prognosis, natural history, and risk of recurrence of the disease in the fetus. The management of cases of pregnancy involving fetal skeletal anomalies depends on three factors: gestational age at the time of diagnosis, severity of the disease, and the decisions made by the parents. When a skeletal anomaly is detected, the whole fetus should be evaluated in order to identify other associated anomalies. The presence or absence of findings on an MRI examination of the fetus using MRI can help determine whether the skeletal anomaly is independent of or associated with other anomalies. This ability to detect anomalies of the fetus is a major contribution of MRI.

## CONCLUSION

In summary, the modified VIBE MRI sequence enabled detailed characterization of the fetal skeleton via 3D reconstructions of the images acquired, allowing the complete evaluation of the fetal skeleton. However, the proposed sequence has one limitation: it shows inadequate accuracy while evaluating the extremities of the fetus. Therefore, improvements to the sequence should be addressed in future studies.
